# Exploring Visual Discrimination and Performance Adaptation in First-League Futsal Players via LUMMICS

**DOI:** 10.3390/vision10020023

**Published:** 2026-04-23

**Authors:** Bruno Monteiro, Ana Roque, Henrique Nacimento, Clara Martinez-Perez

**Affiliations:** 1 Óptica Lentesdecontacto.pt, Av. João Crisóstomo, 38-B, 1050-127 Lisboa, Portugal; bruno.monteiro@lentesdecontacto.pt; 2 School of Administration, Engineering and Aeronautics (EGEA), Instituto Superior de Educação e Ciências de Lisboa (ISEC Lisboa), Alameda das Linhas de Torres, 179, 1750-142 Lisboa, Portugal; ana.roque@iseclisboa.pt (A.R.); henrique.nascimento@iseclisboa.pt (H.N.); 3 Applied Physics Department (Optometry Area), Facultade de Óptica e Optometría, Universidade de Santiago de Compostela, 15705 Santiago de Compostela, Spain

**Keywords:** futsal, visual discrimination, reaction time, LUMMICS

## Abstract

Background/Objectives: Perceptual–cognitive abilities such as visual discrimination, reaction time, and attentional control are important for performance in dynamic sports. However, evidence remains limited regarding how simplified visual tasks capture performance variability and dynamics under repeated exposure. This study examined session-to-session performance changes and individual trajectories in a programmable visual discrimination task of increasing complexity in elite futsal players. Methods: An exploratory repeated-measures study was conducted with ten first-league futsal players. Participants completed between six and ten sessions of a color-cue visual discrimination task (“Follow the Color”) under one- and two-stimulus conditions. Outcomes included correct responses, errors, and reaction time per session. A total of 465 observations were analyzed using linear mixed-effects models to assess changes across sessions and the influence of task complexity. Individual analyses were also performed to explore player-specific trajectories. Results: Mean session accuracy was 63.8 ± 9.8 correct responses, with a mean error rate of 3.6 ± 6.1 and a mean reaction time of 0.63 ± 0.15 s. Error rates declined significantly across sessions (β = −0.008, *p* < 0.001), while reaction time improved modestly (β = −0.00011, *p* = 0.025). Correct responses showed a small negative trend over time. Increased task complexity was associated with fewer correct responses, higher error rates, and slower reaction times (all *p* < 0.001). Conclusions: This programmable visual discrimination task captured variability in visuomotor responses under controlled conditions and may support monitoring of performance dynamics in sports vision research.

## 1. Introduction

Over the past two decades, sport vision (SV) has emerged as an important area of research aimed at understanding how visual and perceptual–cognitive processes contribute to athletic performance. This field integrates knowledge from optometry, neuroscience, psychology, and sports science, and is based on the premise that successful performance depends not only on physical conditioning or technical skills, but also on the efficient processing of visual information and its translation into timely and coordinated motor responses [1,2]. The well-known assertion that “the eyes guide the body” reflects a concept that is increasingly supported by empirical evidence, highlighting the central role of the visual system in anticipation, decision-making, and motor execution during sports practice [3].

A key distinction within sport vision research is that between sight and vision. While sight refers primarily to static visual acuity, vision encompasses a broader set of dynamic processes, including visual discrimination, attention, perception–action coupling, and visuomotor coordination [2,4]. These functions operate continuously in real time and are particularly critical in dynamic team sports. In futsal, for example, players must simultaneously monitor the ball, teammates, and opponents, anticipate rapidly evolving play situations, and adjust motor responses within very short time windows [5,6,7,8,9]. Such demands place a high load on visual attention, reaction time, and perceptual decision-making.

Several visual skills have been identified as relevant to sports performance, including hand–eye coordination, peripheral awareness, dynamic visual acuity, depth perception (stereopsis), visual attention, and reaction time [7,10,11,12,13]. As a result, sport vision training programs have incorporated a wide variety of exercises designed to stimulate these abilities, ranging from oculomotor tasks on light panels to computer-based paradigms, virtual environments, and stroboscopic training systems [14,15,16,17]. However, despite this growing interest, evidence supporting consistent transfer of such training to real competitive performance remains limited or inconclusive [18,19,20].

One of the major methodological challenges in this area is ecological validity. Many experimental tasks used in sport vision research rely on simplified or generic stimuli that only partially reflect the perceptual demands of actual sports environments [21,22]. From an ecological perspective, as proposed by Gibson, perception and action are tightly coupled and can only be fully understood within the context in which they occur [21,22]. Learning, therefore, emerges through repeated interaction with task-relevant information embedded in meaningful environments [22]. At the same time, highly ecological tasks often reduce experimental control, making it difficult to isolate specific perceptual or cognitive components.

Nevertheless, a number of studies have shown that improvements can be observed in specific visual or perceptual tasks following training, even when these tasks are not directly related to the sport being practiced [18,23,24]. Such improvements have been associated with enhanced attentional control, faster stimulus discrimination, and more efficient decision-making under pressure [25,26,27]. In addition, physical exercise and interaction with structured task environments have been linked to broader cognitive benefits, suggesting that simplified perceptual tasks may still capture relevant aspects of visuomotor adaptation and learning [28,29].

In futsal and football-related sports, visual processing acquires particular strategic importance due to the speed of play and the density of players in restricted spaces [8,30,31]. Vision in this context involves not only tracking moving objects but also extracting relevant information from complex visual scenes and responding appropriately under temporal constraints. However, there is still no clear consensus regarding which specific visual abilities are most determinant for performance, nor on the most reliable and reproducible methods to assess them [9,24].

Within this framework, there is growing interest in the use of controlled visual discrimination tasks that allow repeated assessment of performance while maintaining a high degree of standardization. Such tasks do not aim to replicate the full complexity of sports environments, but they provide an opportunity to examine intra-individual variability, performance dynamics, and the influence of task complexity on basic performance metrics such as accuracy, error rate, and reaction time.

Importantly, these approaches are better understood as methodological tools for characterizing performance dynamics rather than as direct proxies of sport-specific performance or training effects.

The present study adopts this approach by implementing a programmable color-cue visual discrimination task (“Follow the Color”) administered repeatedly to first-league futsal players under controlled conditions. Rather than evaluating training efficacy or transfer to sport-specific performance, this investigation focuses on characterizing response patterns across sessions, describing individual trajectories, and examining how increasing task complexity influences response accuracy and latency. By doing so, the study aims to contribute methodological data on the use of simple visual discrimination paradigms for monitoring visuomotor performance in athletic populations.

## 2. Materials and Methods

### 2.1. Study Design

This study employed an exploratory repeated-measures experimental design. Participants completed multiple sessions of a color-based visual discrimination task under two predefined stimulus conditions. The repeated administration of this protocol allowed within-subject assessment of performance over time and comparison between stimulus conditions. The study was conducted at the facilities of Clube Recreativo Leões Porto Salvo between October 2024 and May 2025. All procedures complied with the ethical principles of the Declaration of Helsinki and were approved by the Ethics Committee of the Higher Institute of Education and Sciences of Lisbon (ISEC Lisbon) on 11 September 2023 (approval ID: CE/2023/09/11).

### 2.2. Participant Enrolment

Participants were recruited through voluntary enrolment among active futsal players from the same club. Eligibility criteria included age between 18 and 50 years, normal or corrected-to-normal visual acuity, full upper-limb motor function, and absence of self-reported neurological or psychiatric disorders. Exclusion criteria comprised current use of medication affecting cognitive or motor performance, consumption of alcohol or psychoactive substances within 24 h prior to testing, and acute illness or injury on the day of assessment. Each participant completed between six and ten experimental sessions, depending on availability and adherence to the protocol.

### 2.3. “Follow the Color” Task

Participants performed a visual discrimination task using a system of five programmable reaction lights mounted on a vertical panel. The task was implemented through the LUMMICS^®^ platform (Laboratório de Usabilidade e Medida da Motricidade Integrada no Controlo Sensorial), which allows programmable control of stimulus presentation and automated recording of responses. Four lights were positioned at the corners of a square at approximately shoulder height, with a fifth light located at the center. The task was administered under two stimulus conditions:

- One-stimulus condition.: In each trial, all five lights were illuminated simultaneously. One light displayed the target color (red), while the remaining four displayed distractor colors (e.g., blue, yellow, green, or white). Participants were instructed to touch the red light as quickly as possible. Each session consisted of 60 to 80 randomized trials.
- Two-stimulus condition: In this condition, two target colors (red and green) were used. In each trial, one of the five lights displayed either red or green, while the remaining lights showed distractor colors. Participants were instructed to respond when either target color appeared. Each session also consisted of 60 to 80 randomized trials.

### 2.4. Procedure

Testing was conducted individually in a quiet, well-lit room. Participants stood at a standardized distance from the light panel, which was configured identically across sessions. Prior to the first recorded session, participants completed a brief familiarization trial to ensure comprehension of task instructions. Data from this familiarization phase were not included in the analysis. The order of stimulus conditions was counterbalanced across participants. All stimulus parameters, including timing and randomization, were controlled through the LUMMICS^®^ software interface. Short rest periods were allowed between blocks to minimize fatigue.

### 2.5. Statistical Analysis

All statistical analyses were performed using R (version 4.4.2). The complete R script used for data processing, statistical modeling, and figure generation is provided as Supplementary File S1. The original, unedited statistical output from the software is included as Supplementary File S2, allowing full replication and verification of the results presented. Descriptive statistics were computed for each variable of interest, including number of correct responses, number of errors, and reaction time per session. These included the mean, standard deviation, median, interquartile range, and range. Visual inspection of residuals and Q–Q plots was conducted to evaluate normality assumptions.

Given the variability in the number of trials per session (60–80), outcome measures based on absolute counts were interpreted with caution, and analyses focused on within-subject trends over time. To further address variability in the number of trials per session, a sensitivity analysis was conducted using normalized outcomes, including the proportion of correct responses and errors per trial. These normalized variables were analyzed using the same linear mixed-effects modeling approach.

To assess performance changes over time, linear regression models were fitted with session number as the predictor for each outcome variable. Additionally, linear mixed-effects models were used to account for repeated measures, with fixed effects for session order and stimulus condition, and a random intercept for participant. The general structure of the linear mixed-effects models was specified as follows: outcome ~ session order + stimulus condition + (1|participant), where session order was included as a continuous variable representing temporal progression, stimulus condition as a fixed effect, and participant as a random intercept to account for within-subject correlation across repeated measures. Between- and within-subject variance components were estimated from these models.

Test–retest reliability was evaluated by computing Pearson correlations between performance in Session 1 and Session 10 for both correct responses and reaction time. Confidence intervals were derived using Fisher’s Z transformation. Given the small sample size, these analyses were considered exploratory. A complementary mixed-effects model with session as a random effect was used to estimate session-level variability.

To assess individual performance trajectories, linear models were fitted separately for each participant, regressing correct responses on session number. Slope estimates, standard errors, and *p*-values were extracted and compared across individuals.

Finally, the relationship between reaction time and accuracy was examined using Pearson correlation analysis between average reaction time and number of correct responses across sessions, to explore the presence of a speed–accuracy trade-off. Statistical significance was set at *p* < 0.05.

## 3. Results

### 3.1. Participants

A total of 10 participants completed between 6 and 10 sessions of the “Follow the Color” task, generating a total of 465 observations. Accuracy, measured as the number of correct responses per session, had a mean of 63.8 ± 9.8 (median = 60; IQR = 16; range: 31–80). The number of errors per session showed greater variability, with a mean of 3.6 ± 6.1 (median = 2; IQR = 5; range: 0–49). Regarding response speed, the average reaction time was 0.63 ± 0.15 s (median = 0.588 s; IQR = 0.083 s; range: 0.108–1.386 s).

### 3.2. Intra-Subject Repeated Measures Analysis

**Figure 1:**
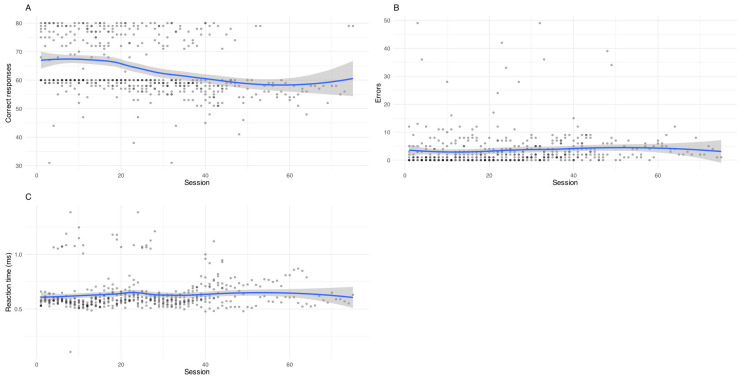
(**A**) Mean correct responses per session; (**B**) Mean errors per session; (**C**) Mean reaction time per session.

The number of correct responses showed a decreasing trend over time (β = −0.017, *p* < 0.001), and participants performed worse under the two-stimulus condition (β = −2.84, *p* < 0.001). Between-subject variability was relatively limited (SD = 4.73), suggesting modest differences in baseline accuracy across participants (Figure 1A).

In contrast, error rates also tended to decrease over time (β = −0.008, *p* < 0.001). Sessions involving more complex stimuli were associated with higher error rates (β = +3.58, *p* < 0.001). Between-subject variance was modest (SD = 1.34), while residual variance was higher (SD = 4.90), suggesting greater within-subject variability (Figure 1B).

The simultaneous reduction in both correct responses and errors does not indicate a straightforward improvement in performance and may instead reflect changes in total responses per session or shifts in response strategy (e.g., more conservative responding). However, this interpretation remains speculative, as response strategies were not directly assessed in the present study.

Regarding reaction speed (Figure 1C), a small decrease in latency was observed (β = −0.00011, *p* = 0.025), suggesting a small but statistically significant reduction in response latency over time. Sessions with two stimuli were associated with significantly slower responses (β = +0.043, *p* < 0.001). Between-subject variance was minimal (SD = 0.041), indicating that changes in latency were primarily explained by fixed effects.

Visual inspection of Figure 1 supports these findings, showing a slight downward trend in correct responses over time, alongside a reduction in error counts and a modest decrease in response latency across sessions.

To further explore the relationship between response speed and accuracy, correlation analysis showed a modest negative association (r = −0.24, *p* < 0.001, 95% CI [−0.32, −0.15]), consistent with the presence of a speed–accuracy trade-off.

**Figure 2:**
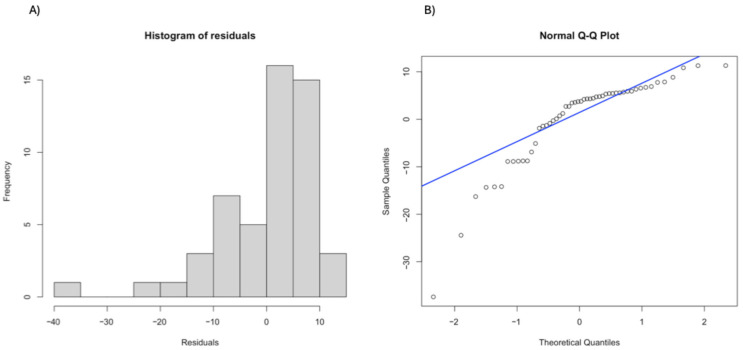
Residual diagnostics for the accuracy model. (**A**) Residuals vs. fitted values. (**B**) Normal Q–Q plot of residuals.

Visual inspection of residuals versus fitted values indicated mild heteroscedasticity (Figure 2A), and Q–Q plots showed minor deviations from normality at the tails (Figure 2B). Figure 2 indicates slight deviations from normality at the distribution tails and some dispersion in residuals, consistent with the mild heteroscedasticity observed. Overall, model assumptions were considered reasonably met, supporting cautious interpretation of the results in this context. When outcomes were normalized by the total number of trials per session, the results provided a more interpretable assessment of performance changes over time. The proportion of correct responses showed a small but statistically significant increase across sessions (β = 0.00144, *p* = 0.0019), while the proportion of errors decreased (β = −0.00144, *p* = 0.0019). These findings suggest that the apparent reduction in absolute correct responses observed in the main analysis may be influenced by variability in the number of trials per session, rather than reflecting a true decline in performance

### 3.3. Individual Performance Trajectories

Among the ten participants, three showed statistically significant positive trends in performance over time, with slope estimates ranging from approximately +0.10 to +0.44 (*p* < 0.05). For example, one participant exhibited a slope of β = +0.439 (SE = 0.190, t = 2.31, *p* = 0.028), suggesting measurable change over time, without a clear indication of performance improvement.

In contrast, one participant showed a significant decline in accuracy over time (β = −0.308, SE = 0.057, t = −5.39, *p* < 0.00001), indicating a gradual decrease in correct responses across sessions. The remaining six participants did not show statistically significant trends, with slope estimates ranging from −0.10 to +0.15 and *p*-values between 0.058 and 0.93, suggesting relatively stable performance over time.

Overall, these findings highlight substantial inter-individual variability in performance trajectories, with no consistent pattern of improvement across participants.

### 3.4. Temporal Consistency Analysis

For the number of correct responses, a moderate correlation was observed between Session 1 and Session 10 (r = 0.63, *p* = 0.049). However, the 95% confidence interval was very wide (0.007–0.903), indicating substantial uncertainty and poor reliability of the estimate. These results should therefore be interpreted with caution.

Complementarily, a linear mixed-effects model with a random intercept for session was fitted to estimate variance across sessions. The model showed negligible between-session variance (SD = 0), while residual variance was high (SD = 9.80), suggesting that most of the variability is attributable to individual or unexplained factors rather than systematic differences across sessions. These findings further support the limited reliability of the measures and should be interpreted cautiously.

In contrast, for reaction time, the correlation between Session 1 and Session 10 was weak and non-significant (r = −0.13, *p* = 0.719), with a wide 95% confidence interval (−0.703 to 0.544), indicating low stability and poor reliability, with considerable variability across individuals.

Overall, the reliability findings should be considered poor and exploratory, given the small sample size and the imprecision reflected in the wide confidence intervals.

### 3.5. Comparison Between Stimulus Conditions

The linear mixed-effects model showed a significant effect of stimulus condition on performance. Participants achieved fewer correct responses during sessions involving two stimuli compared to those with a single stimulus (β = −3.14, *p* < 0.001). The average number of correct responses was 65.4 (SD = 9.33) under the single-stimulus condition and 61.6 (SD = 10.0) under the dual-stimulus condition. Between-subject variability remained moderate (SD = 6.70), and residual variance was also substantial (SD = 7.48), suggesting some within-subject fluctuation across sessions.

**Figure 3:**
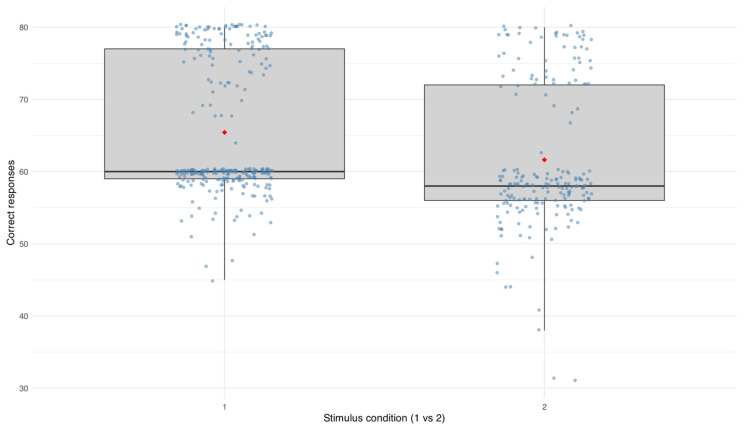
Distribution of correct responses by stimulus condition.

Figure 3 illustrates this pattern, showing the distribution of correct responses across both stimulus conditions. Each dot represents an individual session, with jittered points and mean values overlaid for clarity. The figure confirms this difference, with lower central tendency values and greater dispersion observed under the two-stimulus condition compared to the single-stimulus condition.

## 4. Discussion

This exploratory repeated-measures study assessed the evolution of visual performance in first-league futsal players using a color-based discrimination task (“Follow the Color”). By analyzing accuracy, response speed, and error rates across multiple sessions under controlled stimulus conditions, we identified intra-individual patterns of performance adaptation and task familiarization. Given the small sample size, these findings should be interpreted cautiously and primarily as descriptive trends rather than robust or generalizable effects. These findings are consistent with prior research in the sports vision training (SVT) literature while offering novel contributions in terms of task specificity, individual variability, and stimulus complexity.

Consistent with studies implementing structured SVT programs, our data suggest adaptation over time, characterized by reductions in error rates and small but statistically significant changes in reaction time, alongside a decrease in correct responses. Importantly, the magnitude of the reaction time effect appears extremely small and is unlikely to be meaningful in practical or performance-related contexts. This pattern complicates interpretation and does not support a straightforward improvement effect. Instead, it may reflect shifts in response strategy, such as more conservative or selective responding; however, this interpretation remains speculative and was not directly assessed in the present study. Therefore, these changes are better interpreted as performance adaptation rather than clear evidence of learning or improvement. In the absence of a control condition or baseline stabilization, true learning effects cannot be distinguished from task familiarization or repeated exposure.

Guo et al. [32], in a randomized controlled trial with elite skeet shooters, reported improvements in eye–hand coordination, perception span, and shooting accuracy following a six-week training protocol. While our participants engaged with a less intensive, non-sport-specific task, they exhibited changes in performance patterns that may be consistent with adaptive responses to repeated exposure, particularly under lower stimulus complexity. This suggests that even simplified visual challenges can engage perceptual–motor processes, aligning with the broader principle of neuroplasticity underpinning SVT, although the extent to which such adaptations reflect meaningful performance improvement remains uncertain.

In boxing, Wu et al. [33] found that specific visual abilities, such as depth perception and reaction control, correlated with superior punch performance. Although our study did not assess real-world motor execution, the association between speed and accuracy observed here aligns with their findings: athletes with sharper visual discrimination tend to make faster and more accurate responses. This supports the idea that perceptual skills are foundational to motor execution and may reflect perceptual processes that are relevant to motor execution, although no direct inference can be made regarding sport-specific performance.

Furthermore, the presence of a speed–accuracy trade-off in our task echoes patterns described in other SVT contexts. Appelbaum & Erickson [16], as well as studies reviewed by Lochhead et al. [34], emphasize that optimal athletic performance is not solely dependent on speed or accuracy but rather on the balance between them. Our findings fit within this conceptual framework, highlighting the cognitive control demands imposed by dual-target tasks and the inherent performance compromises they generate. This trade-off may reflect adaptive response strategies under increasing task demands, whereby participants prioritize speed over accuracy, or vice versa, depending on attentional and decisional constraints; however, this interpretation is exploratory and underlying mechanisms cannot be determined from the present data.

Despite these convergences, several distinctions are noteworthy. First, unlike intervention-based studies such as that of Clark et al. [35], where vision training was embedded into pre-season football routines and linked to a measurable reduction in concussion incidence, our task was isolated from real-world sport situations. The “Follow the Color” protocol lacks the spatial and kinesthetic complexity of field-based activities, which may limit ecological transfer. This contrast underscores the importance of context-rich training for translating perceptual gains into performance outcomes, particularly in contact sports. Formenti et al. [36] further explored this issue, comparing cognitive gains in athletes undergoing non-specific vision training versus traditional volleyball practice. Their findings revealed that while visual-cognitive skills improved in the vision training groups, actual sport-specific skill gains were greater in the control group. Similarly, our participants exhibited changes in general task metrics, but the absence of football-related elements, such as spatial tracking, body coordination, or decision-making under dynamic pressure, raises questions about the transferability of these improvements to match play. Another critical difference lies in interindividual variability. In our study, only a few participants showed significant improvements over time, while others remained stable or declined slightly. This pattern parallels findings from Schwab & Memmert [20], who noted limited transfer in hockey players following generic vision training, with gains often restricted to the specific visual tasks practiced. The heterogeneity in performance trajectories may reflect differences in baseline abilities, attentional engagement, or motivational factors, variables that are often underreported in SVT studies.

Moreover, the limited test–retest reliability observed for reaction time contrasts with the more robust reliability of accuracy scores. This indicates that response latency may be more sensitive to transient cognitive states, such as fatigue or distraction. Studies such as those by Poltavski & Biberdorf [37] and Laby et al. [38] have shown that reaction time is a dynamic and context-sensitive measure, often improving only in highly immersive or physically integrated training environments. Our findings reinforce the notion that isolated reaction tasks may not yield stable individual baselines without additional motor or sensory engagement. From a practical perspective, these findings suggest that the task should be used with caution for individual-level monitoring, particularly when interpreting changes across sessions. The observed variability may limit its usefulness for precise tracking of individual performance, although it may still provide informative insights at a group level or for exploratory assessments of performance dynamics.

From a methodological perspective, the task employed in this study bridges elements of component skill training (e.g., color discrimination, speeded response) and perceptual-cognitive training (e.g., go/no-go decision-making), as proposed by Lochhead et al. [34]. However, it does not qualify as “naturalistic” training due to the absence of real-time sport stimuli or physical execution. While this design offers experimental control and replicability, it also limits the ecological richness required for robust transfer to athletic contexts. Nonetheless, our findings suggest that even tasks with reduced motor complexity can capture relevant markers of cognitive flexibility, adaptation, and performance variability. In this sense, the “Follow the Color” protocol may be better understood primarily as a monitoring and research tool, rather than a screening instrument or training intervention. Its main contribution lies in providing a standardized and reproducible framework for assessing visuomotor dynamics over time, particularly in capturing intra-individual variability, adaptation patterns, and responses to changes in task complexity. In applied contexts, this approach may be useful for longitudinal performance monitoring, identification of atypical response profiles, or integration into broader assessment protocols in sports vision and neuro-optometric settings. However, it is not intended to predict or directly assess sport-specific or on-field performance.

One strength of this study is its repeated-measures design, which enabled the characterization of individual performance trajectories over time. The inclusion of both simple and complex stimulus conditions provided insight into how cognitive load modulates performance. Statistical rigor was supported by the use of linear mixed-effects models, allowing appropriate handling of within- and between-subject variability. Additionally, the standardized and controlled experimental setup minimized external confounding factors. The observation of measurable performance changes in a non-immersive, low-resource environment suggests that simplified paradigms may still provide meaningful information about visuomotor adaptation, with potential implications for scalable assessment tools.

### 4.1. Limitations

As an exploratory study, several limitations should be considered when interpreting the findings. The small sample size and recruitment from a single futsal club limit generalizability and reduce statistical power, particularly for detecting subtle effects or between-subject differences. Although the repeated-measures design generated a relatively large number of observations, the effective sample size is determined by the number of participants (n = 10), and therefore the inferential power remains limited. Accordingly, the results should be interpreted as descriptive and hypothesis-generating rather than confirmatory.

The absence of a control or comparison group precludes causal inference and prevents isolation of task-related effects from potential confounders such as test familiarity, attentional variability, or motivation across sessions. In addition, the lack of comparison with non-athlete populations limits the ability to determine whether the observed patterns are specific to trained athletes or reflect more general visuomotor processes. Although the repeated-measures design enables within-subject analysis, it does not allow definitive conclusions regarding training effects.

The visual discrimination task was conducted in a highly controlled and simplified environment. While this facilitates standardization and precise measurement, it inherently limits ecological validity. The task does not replicate sport-specific perceptual cues, full-body motor responses, or decision-making demands typical of futsal competition; therefore, no direct conclusions can be drawn regarding transfer to on-court performance.

Additionally, participants completed a variable number of sessions (six to ten), and the number of trials per session ranged from 60 to 80. This variability introduces heterogeneity in exposure and may influence longitudinal estimates, particularly when outcomes are expressed as absolute counts rather than normalized measures.

Finally, reaction time measures may be sensitive to transient cognitive states and system-related factors, which could contribute to the limited temporal consistency observed for this outcome.

On the other hand, the analytical approach assumed linear changes over sessions, as a simplifying assumption to facilitate modeling and interpretation. However, learning and performance adaptation are often non-linear processes, and more complex trajectories (e.g., plateau effects or rapid early improvements) may be present but were not explored in the present study. This should be considered when interpreting temporal trends.

### 4.2. Future Directions

Building on these findings, future studies should explore the integration of simplified visual tasks like “Follow the Color” into sport-specific drills and ecologically valid dual-task environments—for example, combining ball control, decision-making under pressure, and visual discrimination in dynamic settings. Embedding such tasks into regular training sessions may enhance the development of cognitive-motor coordination and attentional flexibility in real-time game contexts. Expanding research to include larger, more diverse samples, encompassing different levels of expertise, age groups, and sports modalities, would allow for more robust generalizability and subgroup analyses.

From a practical perspective, incorporating wearable sensors (e.g., inertial measurement units, heart rate monitors) and eye-tracking technologies could provide valuable real-time feedback on motor execution, visual attention allocation, and fatigue response. These data may help refine the understanding of visuomotor learning mechanisms and support individualized training prescriptions. In applied research or performance monitoring contexts, especially in sports medicine or neuro-optometric therapy, such protocols could serve as functional assessment tools or as part of cognitive-motor re-education strategies in populations recovering from concussion, visual dysfunction, or motor coordination deficits. By bridging controlled visual assessment with applied movement tasks, these future directions may help operationalize vision training as a routine and evidence-based component of athletic development and clinical care.

## 5. Conclusions

This study suggests that a simple color-based visual discrimination task (“Follow the Color”) can capture intra- and interindividual variability in accuracy, error rates, and response speed under controlled conditions. Across repeated sessions, error rates decreased and reaction times showed modest improvement, while the number of correct responses exhibited a slight downward trend, which may suggest selective adaptation in response strategies, although this interpretation should be considered speculative. Analysis of individual trajectories revealed substantial heterogeneity, with some participants showing positive trends over time and others showing stable or declining patterns. These findings highlight the importance of considering individual response profiles when interpreting repeated visuomotor assessments. Additionally, increased task complexity, operationalized through the introduction of a second target stimulus, negatively affected both accuracy and response latency, underscoring the impact of cognitive load on performance. Accuracy measures showed moderate temporal consistency across sessions, whereas reaction time exhibited considerable variability, likely reflecting sensitivity to transient cognitive or attentional states. Taken together, these results support the use of the “Follow the Color” task, particularly in its programmable and standardized LUMMICS^®^ format, as a controlled methodological tool for exploring visuomotor response patterns and individual differences. However, these findings should be interpreted within the context of a simplified stimulus–response paradigm and do not establish direct relevance to sport-specific performance or on-field demands.

## Data Availability

The original contributions presented in this study are included in the article. Further inquiries can be directed to the corresponding author.

## Supplementary Materials

The following supporting information can be downloaded at: https://www.mdpi.com/article/10.3390/vision10020023/s1, Supplementary File S1: R script for data preprocessing and mixed-effects modelling; Supplementary File S2: R script for model fitting and statistical outputs.
