# Supplementary material for: Exploring Visual Discrimination and Performance Adaptation in First-League Futsal Players via LUMMICS
Source: Vision (Basel). 2026 Apr 23;10(2):23. doi: 10.3390/vision10020023 (PMC13214638; doi:10.3390/vision10020023)
Supplement: Supplementary file 1 [file vision-10-00023-s001.zip › Supplementary File S1.pdf]

```
############################################################
# Required libraries
############################################################
library(dplyr)
library(lme4)
library(lmerTest)
library(tidyr)
library(broom)
library(lubridate)

############################################################
# 1. Load and preprocess data
############################################################
data <- Dados_final

# Convertir fechas correctamente
if (is.numeric(data$Fecha)) {
  data$Fecha <- as.Date(data$Fecha, origin = "1899-12-30")
} else {
  data$Fecha <- suppressWarnings(ymd(data$Fecha))
  if (all(is.na(data$Fecha))) data$Fecha <- dmy(data$Fecha)
}

# Variables numéricas y factores
data$Erros <- as.numeric(gsub("[^0-9.]", "", data$Erros))
data$Estímulos <- as.factor(data$Estímulos)

# Crear variable Sesion y Fecha_num
data <- data %>%
  arrange(Nombre, Fecha) %>%
  group_by(Nombre) %>%
  mutate(Sesion = row_number(),
         Fecha_num = as.numeric(Fecha)) %>%
  ungroup()

############################################################
# 2. Descriptive statistics
############################################################
n_part <- n_distinct(data$Nombre)
n_obs <- nrow(data)

desc_correctos <- data %>% summarise(mean=mean(Correctos), sd=sd(Correctos),
                                     median=median(Correctos), IQR=IQR(Correctos),
                                     min=min(Correctos), max=max(Correctos))

desc_errores <- data %>% summarise(mean=mean(Erros), sd=sd(Erros),
                                   median=median(Erros), IQR=IQR(Erros),
                                   min=min(Erros), max=max(Erros))

desc_velocidad <- data %>% summarise(mean=mean(`Velocidad reacción (ms)`),
sd=sd(`Velocidad reacción (ms)`),
                                     median=median(`Velocidad reacción (ms)`),
IQR=IQR(`Velocidad reacción (ms)`),
                                     min=min(`Velocidad reacción (ms)`),
max=max(`Velocidad reacción (ms)`))

############################################################
# 3. Linear mixed-effects models with Date_num and Stimulus
############################################################
data_filtered <- data %>% filter(!is.na(Fecha_num))

m_corr <- lmer(Correctos ~ Estímulos + Fecha_num + (1 | Nombre), data = data_filtered)
m_err  <- lmer(Erros ~ Estímulos + Fecha_num + (1 | Nombre), data = data_filtered)
m_vel  <- lmer(`Velocidad reacción (ms)` ~ Estímulos + Fecha_num + (1 | Nombre), data =
data_filtered)

coef_corr <- summary(m_corr)$coefficients
coef_err <- summary(m_err)$coefficients
```

```
coef_vel <- summary(m_vel)$coefficients

sd_corr <- as.data.frame(VarCorr(m_corr))$sdcor[1]
sd_err <- as.data.frame(VarCorr(m_err))$sdcor[1]
sd_vel <- as.data.frame(VarCorr(m_vel))$sdcor[1]

############################################################
# 4. Correlation Speed-Accuracy (mean by session)
############################################################
vel_prec <- data %>%
  group_by(Nombre, Sesion) %>%
  summarise(mean_correctos = mean(Correctos),
            mean_velocidad = mean(`Velocidad reacción (ms)`),
            .groups = "drop")

cor_vp <- cor.test(vel_prec$mean_velocidad, vel_prec$mean_correctos)

############################################################
# 5. Test-Retest: Session 1 vs 10 (only participants with ≥10 sessions)
############################################################
tt_data <- data %>%
  group_by(Nombre) %>%
  filter(max(Sesion) >= 10) %>%
  ungroup()

tt_corr <- tt_data %>%
  filter(Sesion %in% c(1, 10)) %>%
  select(Nombre, Sesion, Correctos) %>%
  pivot_wider(names_from = Sesion, values_from = Correctos, names_prefix = "Sesion_")
%>%
  filter(!is.na(Sesion_1) & !is.na(Sesion_10))

cor_tt_corr <- cor.test(tt_corr$Sesion_1, tt_corr$Sesion_10)

tt_vel <- tt_data %>%
  filter(Sesion %in% c(1, 10)) %>%
  select(Nombre, Sesion, `Velocidad reacción (ms)`) %>%
  pivot_wider(names_from = Sesion, values_from = `Velocidad reacción (ms)`,
names_prefix = "Sesion_") %>%
  filter(!is.na(Sesion_1) & !is.na(Sesion_10))

cor_tt_vel <- cor.test(tt_vel$Sesion_1, tt_vel$Sesion_10)

############################################################
# 6. Individual learning slopes for each participant
############################################################
ind_slopes <- data %>%
  group_by(Nombre) %>%
  do(tidy(lm(Correctos ~ Fecha_num, data = .))) %>%
  filter(term == "Fecha_num") %>%
  select(Nombre, estimate, std.error, statistic, p.value)

mejoran <- ind_slopes %>% filter(p.value < 0.05 & estimate > 0)
empeoran <- ind_slopes %>% filter(p.value < 0.05 & estimate < 0)

############################################################
# 7. Variance between and within sessions
############################################################
m_var_sesion <- lmer(Correctos ~ (1 | Nombre) + (1 | Sesion), data = data_filtered)
var_sesion <- as.data.frame(VarCorr(m_var_sesion))

############################################################
# 8. Print full results
############################################################
cat("Participants:", n_part, "\n")
cat("Observations:", n_obs, "\n\n")

cat("Correct:\n"); print(desc_correctos)
```

```
cat("\nErrors:\n"); print(desc_errores)
cat("\nReaction time:\n"); print(desc_velocidad)

cat("\nIntra-subject repeated measures analysis (Model Coefficients):\n")
cat("Correctos - Pendiente Fecha_num:", coef_corr["Fecha_num", "Estimate"],
    "p =", coef_corr["Fecha_num", "Pr(>|t|)"], "\n")
cat("Errors - Date_num slope:", coef_err["Fecha_num", "Estimate"],
    "p =", coef_err["Fecha_num", "Pr(>|t|)"], "\n")
cat("Reaction time - Date_num slope:", coef_vel["Fecha_num", "Estimate"],
    "p =", coef_vel["Fecha_num", "Pr(>|t|)"], "\n")

cat("Correct - Stimulus2 effect:", coef_corr["Estímulos2", "Estimate"],
    "p =", coef_corr["Estímulos2", "Pr(>|t|)"], "\n")
cat("Errors - Stimulus2 effect:", coef_err["Estímulos2", "Estimate"],
    "p =", coef_err["Estímulos2", "Pr(>|t|)"], "\n")
cat("Reaction time - Stimulus2 effect:", coef_vel["Estímulos2", "Estimate"],
    "p =", coef_vel["Estímulos2", "Pr(>|t|)"], "\n")

cat("Random SD Correct:", sd_corr, "\n")
cat("Random SD Errors:", sd_err, "\n")
cat("Random SD Reaction time:", sd_vel, "\n")

cat("\nSpeed-Accuracy correlation (r):", cor_vp$estimate, "p =", cor_vp$p.value, "\n")

cat("\nTest-Retest Correct (Session 1 vs 10):\n")
cat("r =", cor_tt_corr$estimate, "p =", cor_tt_corr$p.value, "\n")

cat("\nTest-Retest Reaction time (Session 1 vs 10):\n")
cat("r =", cor_tt_vel$estimate, "p =", cor_tt_vel$p.value, "\n")

cat("\nIndividual Learning Trajectories (Slopes) - Mejoran:\n")
print(mejoran)

cat("\nIndividual Learning Trajectories (Slopes) - Empeoran:\n")
print(empeoran)

cat("\nBetween-session variance (SD):", sqrt(var_sesion$vcov[var_sesion$grp ==
"Sesion"]), "\n")
cat("Residual variance (SD):", sqrt(var_sesion$vcov[var_sesion$grp == "Residual"]),
"\n")
```
