# Supplementary material for: Exploring Visual Discrimination and Performance Adaptation in First-League Futsal Players via LUMMICS
Source: Vision (Basel). 2026 Apr 23;10(2):23. doi: 10.3390/vision10020023 (PMC13214638; doi:10.3390/vision10020023)
Supplement: Supplementary file 1 [file vision-10-00023-s001.zip › Supplementary File S2.pdf]

```
> ############################################################
> # Required libraries
> ############################################################
> library(dplyr)
> library(lme4)
> library(lmerTest)
> library(tidyr)
> library(broom)
> library(lubridate)
>
> ############################################################
> # 1. Load and preprocess data
> ############################################################
> data <- Dados_final
>
> # Convertir fechas correctamente
> if (is.numeric(data$Fecha)) {
+   data$Fecha <- as.Date(data$Fecha, origin = "1899-12-30")
+ } else {
+   data$Fecha <- suppressWarnings(ymd(data$Fecha))
+   if (all(is.na(data$Fecha))) data$Fecha <- dmy(data$Fecha)
+ }
>
> # Variables numéricas y factores
> data$Erros <- as.numeric(gsub("[^0-9.]", "", data$Erros))
> data$Estímulos <- as.factor(data$Estímulos)
>
> # Crear variable Sesion y Fecha_num
> data <- data %>%
+   arrange(Nombre, Fecha) %>%
+   group_by(Nombre) %>%
+   mutate(Sesion = row_number(),
+          Fecha_num = as.numeric(Fecha)) %>%
+   ungroup()
>
> ############################################################
> # 2. Descriptive statistics
> ############################################################
> n_part <- n_distinct(data$Nombre)
> n_obs <- nrow(data)
>
> desc_correctos <- data %>% summarise(mean=mean(Correctos), 
sd=sd(Correctos),
+                                      median=median(Correctos), 
IQR=IQR(Correctos),
+                                      min=min(Correctos), 
max=max(Correctos))
>
> desc_errores <- data %>% summarise(mean=mean(Erros), sd=sd(Erros),
+                                    median=median(Erros), 
IQR=IQR(Erros),
+                                    min=min(Erros), max=max(Erros))
>
> desc_velocidad <- data %>% summarise(mean=mean(`Velocidad reacción
```

```
(ms)`), sd=sd(`Velocidad reacción (ms)`),
+                                      median=median(`Velocidad
reacción (ms)`), IQR=IQR(`Velocidad reacción (ms)`),
+                                      min=min(`Velocidad reacción
(ms)`), max=max(`Velocidad reacción (ms)`))
>
> ############################################################
> # 3. Linear mixed-effects models with Date_num and Stimulus
> ############################################################
> data_filtered <- data %>% filter(!is.na(Fecha_num))
>
> m_corr <- lmer(Correctos ~ Estímulos + Fecha_num + (1 | Nombre),
data = data_filtered)
> m_err  <- lmer(Erros ~ Estímulos + Fecha_num + (1 | Nombre), data
= data_filtered)
> m_vel  <- lmer(`Velocidad reacción (ms)` ~ Estímulos + Fecha_num +
(1 | Nombre), data = data_filtered)
>
> coef_corr <- summary(m_corr)$coefficients
> coef_err <- summary(m_err)$coefficients
> coef_vel <- summary(m_vel)$coefficients
>
> sd_corr <- as.data.frame(VarCorr(m_corr))$sdcor[1]
> sd_err <- as.data.frame(VarCorr(m_err))$sdcor[1]
> sd_vel <- as.data.frame(VarCorr(m_vel))$sdcor[1]
>
> ############################################################
> # 4. Correlation Speed–Accuracy (mean by session)
> ############################################################
> vel_prec <- data %>%
+   group_by(Nombre, Sesion) %>%
+   summarise(mean_correctos = mean(Correctos),
+             mean_velocidad = mean(`Velocidad reacción (ms)`),
+             .groups = "drop")
>
> cor_vp <- cor.test(vel_prec$mean_velocidad,
vel_prec$mean_correctos)
>
> ############################################################
> # 5. Test–Retest: Session 1 vs 10 (only participants with ≥10
sessions)
> ############################################################
> tt_data <- data %>%
+   group_by(Nombre) %>%
+   filter(max(Sesion) >= 10) %>%
+   ungroup()
>
> tt_corr <- tt_data %>%
+   filter(Sesion %in% c(1, 10)) %>%
+   select(Nombre, Sesion, Correctos) %>%
+   pivot_wider(names_from = Sesion, values_from = Correctos,
names_prefix = "Sesion_") %>%
+   filter(!is.na(Sesion_1) & !is.na(Sesion_10))
>
```

```
> cor_tt_corr <- cor.test(tt_corr$Sesion_1, tt_corr$Sesion_10)
>
> tt_vel <- tt_data %>%
+   filter(Sesion %in% c(1, 10)) %>%
+   select(Nombre, Sesion, `Velocidad reacción (ms)`) %>%
+   pivot_wider(names_from = Sesion, values_from = `Velocidad
reacción (ms)`, names_prefix = "Sesion_") %>%
+   filter(!is.na(Sesion_1) & !is.na(Sesion_10))
>
> cor_tt_vel <- cor.test(tt_vel$Sesion_1, tt_vel$Sesion_10)
>
> ############################################################
> # 6. Individual learning slopes for each participant
> ############################################################
> ind_slopes <- data %>%
+   group_by(Nombre) %>%
+   do(tidy(lm(Correctos ~ Fecha_num, data = .))) %>%
+   filter(term == "Fecha_num") %>%
+   select(Nombre, estimate, std.error, statistic, p.value)
>
> mejoran <- ind_slopes %>% filter(p.value < 0.05 & estimate > 0)
> empeoran <- ind_slopes %>% filter(p.value < 0.05 & estimate < 0)
>
> ############################################################
> # 7. Variance between and within sessions
> ############################################################
> m_var_sesion <- lmer(Correctos ~ (1 | Nombre) + (1 | Sesion), data
= data_filtered)
boundary (singular) fit: see help('isSingular')
> var_sesion <- as.data.frame(VarCorr(m_var_sesion))
>
> ############################################################
> # 8. Print full results
> ############################################################
> cat("Participants:", n_part, "\n")
Participants: 10
> cat("Observations:", n_obs, "\n\n")
Observations: 465

>
> cat("Correct:\n"); print(desc_correctos)
Correct:
# A tibble: 1 × 6
   mean    sd median   IQR   min   max
  <dbl> <dbl>  <dbl> <dbl> <dbl> <dbl>
1  63.8  9.80     60    16    31    80
> cat("\nErrors:\n"); print(desc_errores)

Errors:
# A tibble: 1 × 6
   mean    sd median   IQR   min   max
  <dbl> <dbl>  <dbl> <dbl> <dbl> <dbl>
1  3.57  6.08      2     5     0    49
> cat("\nReaction time:\n"); print(desc_velocidad)
```

```
Reaction time:
# A tibble: 1 × 6
   mean    sd median    IQR   min   max
  <dbl> <dbl>  <dbl>  <dbl> <dbl> <dbl>
1 0.632 0.146  0.588 0.0830 0.108  1.39
>
> cat("\nIntra-subject repeated measures analysis (Model
Coefficients):\n")

Intra-subject repeated measures analysis (Model Coefficients):
> cat("Correctos - Pendiente Fecha_num:", coef_corr["Fecha_num",
"Estimate"],
+     "p =", coef_corr["Fecha_num", "Pr(>|t|)"], "\n")
Correctos - Pendiente Fecha_num: -0.01694459 p = 8.930002e-09
> cat("Errors - Date_num slope:", coef_err["Fecha_num", "Estimate"],
+     "p =", coef_err["Fecha_num", "Pr(>|t|)"], "\n")
Errors - Date_num slope: -0.008011505 p = 3.902234e-05
> cat("Reaction time - Date_num slope:", coef_vel["Fecha_num",
"Estimate"],
+     "p =", coef_vel["Fecha_num", "Pr(>|t|)"], "\n")
Reaction time - Date_num slope: -0.0001098055 p = 0.0248086
>
> cat("Correct - Stimulus2 effect:", coef_corr["Estímulos2",
"Estimate"],
+     "p =", coef_corr["Estímulos2", "Pr(>|t|)"], "\n")
Correct - Stimulus2 effect: -2.844561 p = 4.258542e-05
> cat("Errors - Stimulus2 effect:", coef_err["Estímulos2",
"Estimate"],
+     "p =", coef_err["Estímulos2", "Pr(>|t|)"], "\n")
Errors - Stimulus2 effect: 3.583641 p = 2.987014e-12
> cat("Reaction time - Stimulus2 effect:", coef_vel["Estímulos2",
"Estimate"],
+     "p =", coef_vel["Estímulos2", "Pr(>|t|)"], "\n")
Reaction time - Stimulus2 effect: 0.04332881 p = 0.0009229966
>
> cat("Random SD Correct:", sd_corr, "\n")
Random SD Correct: 4.730926
> cat("Random SD Errors:", sd_err, "\n")
Random SD Errors: 1.340233
> cat("Random SD Reaction time:", sd_vel, "\n")
Random SD Reaction time: 0.04079776
>
> cat("\nSpeed-Accuracy correlation (r):", cor_vp$estimate, "p =",
cor_vp$p.value, "\n")

Speed-Accuracy correlation (r): -0.2381366 p = 2.033353e-07
>
> cat("\nTest-Retest Correct (Session 1 vs 10):\n")

Test-Retest Correct (Session 1 vs 10):
> cat("r =", cor_tt_corr$estimate, "p =", cor_tt_corr$p.value, "\n")
r = 0.634119 p = 0.04895371
>
```

```
> cat("\nTest–Retest Reaction time (Session 1 vs 10):\n")

Test–Retest Reaction time (Session 1 vs 10):
> cat("r =", cor_tt_vel$estimate, "p =", cor_tt_vel$p.value, "\n")
r = -0.130713 p = 0.7189009
>
> cat("\nIndividual Learning Trajectories (Slopes) - Mejoran:\n")

Individual Learning Trajectories (Slopes) - Mejoran:
> print(mejoran)
# A tibble: 3 × 5
# Groups:   Nombre [3]
  Nombre   estimate std.error statistic p.value
  <chr>       <dbl>     <dbl>     <dbl>   <dbl>
1 Lara      0.00603   0.00282      2.14 0.0376
2 Martim    0.117     0.0470       2.48 0.0211
3 Martim_F  0.0113    0.00366      3.08 0.00365
>
> cat("\nIndividual Learning Trajectories (Slopes) - Empeoran:\n")

Individual Learning Trajectories (Slopes) - Empeoran:
> print(empeoran)
# A tibble: 2 × 5
# Groups:   Nombre [2]
  Nombre    estimate std.error statistic  p.value
  <chr>        <dbl>     <dbl>     <dbl>    <dbl>
1 Andre      -0.0413   0.00492     -8.40 8.18e-12
2 Rodrigo_P  -0.0389   0.00566     -6.88 1.73e- 8
>
> cat("\nBetween-session variance (SD):",
sqrt(var_sesion$vcov[var_sesion$grp == "Sesion"]), "\n")

Between-session variance (SD): 0
> cat("Residual variance (SD):", sqrt(var_sesion$vcov[var_sesion$grp
== "Residual"]), "\n")
Residual variance (SD): 7.113535
```
